# Genes Related to Ion-Transport and Energy Production Are Upregulated in Response to CO_2_-Driven pH Decrease in Corals: New Insights from Transcriptome Analysis

**DOI:** 10.1371/journal.pone.0058652

**Published:** 2013-03-27

**Authors:** Jeremie Vidal-Dupiol, Didier Zoccola, Eric Tambutté, Christoph Grunau, Céline Cosseau, Kristina M. Smith, Michael Freitag, Nolwenn M. Dheilly, Denis Allemand, Sylvie Tambutté

**Affiliations:** 1 Centre Scientifique de Monaco, Monaco, Monaco; 2 Univ. Perpignan Via Domitia, Ecologie et Evolution des Interactions, UMR 5244, Perpignan, France; 3 CNRS, Ecologie et Evolution des Interactions, UMR 5244, Perpignan, France; 4 Department of Biochemistry and Biophysics, Center for Genome Research and Biocomputing, Oregon State University, Corvallis, Oregon, United States of America; University of Gothenburg, Sweden

## Abstract

Since the preindustrial era, the average surface ocean pH has declined by 0.1 pH units and is predicted to decline by an additional 0.3 units by the year 2100. Although subtle, this decreasing pH has profound effects on the seawater saturation state of carbonate minerals and is thus predicted to impact on calcifying organisms. Among these are the scleractinian corals, which are the main builders of tropical coral reefs. Several recent studies have evaluated the physiological impact of low pH, particularly in relation to coral growth and calcification. However, very few studies have focused on the impact of low pH at the global molecular level. In this context we investigated global transcriptomic modifications in a scleractinian coral (*Pocillopora damicornis*) exposed to pH 7.4 compared to pH 8.1during a 3-week period. The RNAseq approach shows that 16% of our transcriptome was affected by the treatment with 6% of upregulations and 10% of downregulations. A more detailed analysis suggests that the downregulations are less coordinated than the upregulations and allowed the identification of several biological functions of interest. In order to better understand the links between these functions and the pH, transcript abundance of 48 candidate genes was quantified by q-RT-PCR (corals exposed at pH 7.2 and 7.8 for 3 weeks). The combined results of these two approaches suggest that pH≥7.4 induces an upregulation of genes coding for proteins involved in calcium and carbonate transport, conversion of CO_2_ into HCO_3_^−^ and organic matrix that may sustain calcification. Concomitantly, genes coding for heterotrophic and autotrophic related proteins are upregulated. This can reflect that low pH may increase the coral energy requirements, leading to an increase of energetic metabolism with the mobilization of energy reserves. In addition, the uncoordinated downregulations measured can reflect a general trade-off mechanism that may enable energy reallocation.

## Introduction

Coral reefs are key ecosystems characterized by a high level of biodiversity, ecological complexity and primary productivity. Reef-building corals are key species in coral reefs, providing physical and biological foundations for the ecosystem. The ability of corals to build reefs in oligotrophic tropical oceans is mainly explained by the mutualistic association they form with zooxanthellae (photosynthetic dinoflagellates of the genus S*ymbiodinium*), which produce autotrophically most of the energy required for the calcification process [1].

Global climate change is impacting on coral reefs, with approximately 19% of reefs worldwide being permanently degraded, 15% showing symptoms of imminent collapse, and another 20% at risk of becoming critically affected in the next few decades [2]. This alarming level of reef degradation is mainly due to an increase in frequency and intensity of natural and anthropogenic disturbances affecting reefs [3]. Among, these disturbances is ocean pH decrease, also termed “ocean acidification” [4], [5].

Several experimental studies have shown that low pH decreases the net calcification rate and skeletal growth of various coral species [6]–[15]. However, in other studies no effects on gross calcification (or an increase) have been reported [16]–[18]. In ecosystems naturally subject to low pH conditions (e.g. CO_2_ vents), coral net calcification has been reported to not decrease, or to decrease only slightly [19], [20], but at the ecosystem level low pH induces marked community changes [19]. In addition to its impact on calcification rates, low pH has been shown to: (i) modify the composition, size, shape and orientation of aragonite crystals in primary polyps [7]; and (ii) increase the coral tissue biomass [11], [21]. Contradictory findings have been reported in relation to the effects of low pH on the symbiont component of the coral holobiont. These include findings of a decrease in the density of zooxanthellae [17], [22], and effects on their chlorophyll concentration [11] and photosynthetic efficiency [9]. On the other hand, these results have been challenged in recent studies [9], [14], [16]. To date only 2 recent works have explored the transcriptomic response of corals exposed to low pH: a whole transcriptomic analysis on early life history stages [23] and a cDNA microarrays analysis on adults [17]. Both of these studies were performed on the coral *Acropora millepora* which phylogenetically belongs to the coral “complex” clade which has a less heavily calcified skeletons, and a more porous construction of corallite walls than corals belonging to the “robust” clade [24], [25].

The aim of the present study was to initiate a whole-transcriptome analysis in the adult scleractinian tropical coral *Pocillopora damicornis* exposed to low pH. Such whole-transcriptome analysis has already successfully been used in studies on the impact of global climate change on coastal organisms [23], [26]–[28]. It is also an interesting prerequisite for studies that will target specific biological functions or phenotypic plasticity across populations facing different pH conditions. Finally, RNAseq experiments offer the possibility to highlight unexpected physiological pathways. In the present study we choose to work on the scleractinian tropical coral *P. damicornis* for several reasons: i) it is a major reef-building coral widely distributed in the Indo-Pacific ocean [29]; ii) it is naturally confronted to diverse values of ocean pH, ranging from 7.8 in the particular case of volcanic CO_2_ vents, to 7.9–8.0 in the Eastern tropical Pacific and to 8.1 in other locations [19], [30]; iii) it is known to be highly sensitive to a wide range of natural and anthropogenic disturbances [31]–[33]; iv) it belongs to the “robust” clade which has not yet been studied by transcriptomic approaches [24], [25]. This “robust” clade is characterized by a highly calcified and low porous skeleton compared to corals belonging to the “complex” clade. Taking into account the impact of low pH on calcifying organisms, these last characteristics can lead to different responses to low pH conditions between clades. To address our aims we first assembled the transcriptomes of *P. damicornis* using the result of several RNAseq experiments. In order to maximize the amplitude of the transcriptomic response, an exposure of three weeks to an extreme value of today (volcanic CO_2_ vents) or near future natural variability (lower value on the daily variation scale) of pH (7.4) was used and the transcriptome obtained was compared to the control transcriptome (nubbins maintained for three weeks at a current seawater pH of 8.1). After the identification of the biological functions of interest responding to this low pH, a candidate gene approach (by q-RT-PCR) was performed on mRNA from nubbins exposed to a predicted pH of 7.8 and to a very low pH of 7.2 (3 weeks of exposure).

## Methods

### Coral collection, maintenance, and experimental stress

The *Pocillopora damicornis* (Linnaeus, 1758) isolate used in this study was collected in Lombok, Indonesia (CITES Management Authority, CITES number 06832/VI/SATS/LN/2001-E; Direction de l'Environnement, CITES number 06832/VI/SATS/LN/2001-I). The site of collection corresponds to an usual fringing-reef subjected to the usual day/night pH variation (8.3/7.85) observed on such reefs [34], [35]. *P. damicornis* colonies were then maintained since the year 2001 in an open water aquarium system where pH fluctuates between day and night due to photosynthesis and respiration between maxima and minima values of 8.3 and 7.9 respectively for day and night. For experiments, coral explants (3 cm height, 3 cm diameter) from the same parent colony were left to allow tissues to grow over skeleton (recover) for a period of 1 month prior to use in experiments.

Following recovery, coral nubbins were incubated during 3 weeks into experimental tanks. Temperature (25±0.5°C) was kept constant in each tank using heaters connected to electronic controllers (IKS, Karlsbad, Germany), and seawater was mixed using 2 submersible pumps. Corals received a constant saturating irradiance of 175±10 µmol photons m^−2^ s^−1^ (photoperiod was 12 h∶12 h light/dark) using HQI-10000K metal halide lamps (BLV-Nepturion). They were fed twice a week with *Artemia salina* nauplii in order to reach a natural heterotrophic rate. Tanks, pumps, sensors and electrodes were rigorously cleaned every week to prevent the growth of epiphytic algae and fouling communities or the accumulation of detritus. Water renewal rate in each tank was 60% per hour (continuous water flow).

Carbonate chemistry was manipulated in 3 of 4 tanks by bubbling with CO_2_ to reduce pH to target values and the fourth tank (control tank) was not bubbled with CO_2_ (see values below). pH electrodes and temperature sensors installed in each tank were connected to a pH-stat system (IKS, Karlsbad) that continuously monitored pH (calibrated to NBS scale), temperature and controlled CO_2_ bubbling. On a daily basis, additional pH checks were carried with a Plastogel-Ponsel pH probe (Ponsel), calibrated to pH total scale (pH_T_). Moreover biweekly pH_T_ measurements were made using the indicator dye m-cresol purple (mCP Acros 199250050) adapted from Dickson *et* *al.* [36]; the absorbance was measured using spectrophotometer (UVmc^2^, Safas, Monaco). According to these results, the pH-stat system was adjusted. The control tank was maintained at ambient pH_T_: 8.07±0.07 (396 µatm pCO_2_), the other tanks at pH_T_: 7.75±0.12, (856 µatm pCO_2_)_;_ pH_T_: 7.42±0.21 (2181 µatm pCO_2_)_;_ pH_T_: 7.19±0.18 (3880 µatm pCO_2_). For easier reading, throughout the manuscript, these four values of pH_T_ will be respectively indicated as pH 8.1 (control tank), pH 7.8, pH 7.4 and pH 7.2. After 3 weeks of exposure, 3 nubbins in each treatment tank were sampled at the same time and stored in liquid nitrogen until analyzed.

Water samples were collected in scintillation vials (Wheaton) and stored refrigerated no more than 1 week prior to measurement. Biweekly total alkalinity (TA) was measured via titration with 0.01 N HCl containing 40.7 g NaCl l^−1^ using a Metrohm Titrando 888 dosimat controlled by Tiamo software to perform automated normalized Gran titrations of 1 ml samples. For each sample run, certified seawater reference material supplied by the lab of A.G. Dickson (Scripps Institution of Oceanography) was used to check quality of measurements.

Parameters of carbonate seawater chemistry were calculated from pH_T_, mean AT, temperature, and salinity using the free access CO_2_ Systat package [37] using constants from Mehrbach *et* *al.* [38] as refit by Dickson and Millero [39]. Parameters of carbonate seawater chemistry are given in Table 1 and 2.

**Table 1:** Seawater chemistry (Total alkalinity, pH, TC and *p*CO_2_) obtained from continuously monitored pH.

	Total alkalinity (µmol/kg-SW)		pH		TC (µmol/kg-SW)		*p*CO_2_ (µatm)	
	Mean	SD	Mean	SD	Mean	SD	Mean	SD
**Tank 8.1**	2515.0	18.0	8.07	0.07	2155.33	33.96	396.24	57.2
**Tank 7.8**	2557.0	22.0	7.79	0.08	2319.10	39.82	856.97	182.38
**Tank 7.4**	2498.0	17.0	7.42	0.06	2457.13	22.15	2180.86	323.17
**Tank 7.2**	2542.0	13.0	7.19	0.07	2587.85	27.91	3879.69	654.28

**Table 2:** Seawater chemistry (CO_2_, HCO_3_^−^, CO_3_^−^, Ω Ca^2−^ and Ω Aragonite) obtained from continuously monitored pH.

	CO_2_ (µmol/kg-SW)		HCO_3_^− ^(µmol/kg-SW)		CO_3_^2− ^(µmol/kg-SW)		Ω Ca^2−^		Ω Aragonite	
	Mean	SD	Mean	SD	Mean	SD	Mean	SD	Mean	SD
**Tank 8.1**	11.04	1.59	1887.31	54.52	256.98	22.15	6.03	0.52	3.99	0.34
**Tank 7.8**	23.88	5.08	2142.15	58.77	153.07	24.03	3.59	0.56	2.38	0.37
**Tank 7.4**	60.77	9.00	2325.48	22.27	70.89	9.13	1.66	0.21	1.10	0.14
**Tank 7.2**	108.10	18.23	2436.02	16.46	43.72	6.78	1.03	0.16	0.68	0.11

### cDNA library construction and high-throughput sequencing

We assembled a transcriptome *de novo* from 80-nucleotide paired-end short sequence reads (SSRs) based on 6 lanes of Illumina sequencing. To maximize the coverage of potential transcriptomes, the cDNA from corals exposed to various environmental conditions (pH 7.4 and its dedicated control (see above), thermal stress, bacterial challenge, bacterial infection and a dedicated control for these last conditions [40]) were sequenced (one illumina lane per condition). Despite the recent publication of a reference transcriptome for *P. damicornis* [41] we preferred to assemble *de novo* a reference transcriptome with the SSRs generated by our RNAseq approach rather than map our reads on the one previously published. Indeed, this reference transcriptome (Roche 454 data) was obtained from Hawaiian *P. damicornis*, a population that is geographically very distant from the one we studied (Lombok, Indonesia). Such a distance may lead to a high number of single nucleotide polymorphisms that can skew the mapping step. In addition, the Hawaiian study did not use specimens exposed to low pH, thatpH that could lead to the absence of transcripts or spliced transcripts specifically expressed under such environmental condition.

Total RNA from 3 nubbins per condition was extracted, purified using TRIzol reagent (Invitrogen), and pooled together (one pool per condition) to reach a sufficient amount of biological material for the RNAseq procedure, as described previously [42]. A cDNA library was subsequently constructed following a published protocol [43]. A minimum of 1 µg of cDNA was used to generate each of the paired-end Illumina sequencing libraries. The libraries were prepared using Illumina adapter and PCR primers according to previously published protocols [44], [45]. Libraries with an average insert size of 400–500 bp were isolated and the concentration was adjusted to 10 nM. The samples (7 pM per sample) were loaded into separate channels of an Illumina GAIIx sequencer, and sequenced at the Oregon State University Center for Genome Research and Biocomputing.

### Reference transcriptome assembly and annotation

The SSRs obtained were processed using an Illumina pipeline (RTA1.6_CASAVA1.6), and SSRs from ‘sequence.txt’ files were filtered to select only those SSRs that matched high quality standards. Velvet software version 1.1.04 was subsequently used, evoking velveth with parameters *[k-mer] – fasta – shortPaired*, and velvetg with parameter *-read_trkg yes* [46]. The k-mer value was tested between 31 and 61 and optimized using a subset of data for maximum size of longest contig, average contig length, contig number, n50 value and total transcriptome length. Optima were obtained at k-mer 55 and 57, and we decided for k-mer 55 based on the higher number of included reads. Using the above parameters, 45,596,723 of 131,859,606 SSRs were incorporated into the assembly, producing 102,571 contigs. These served as the input for the *de novo* transcriptome assembler software Oases 0.1.21 [47], using the input parameter *-ins_length 400* and *default parameters -cov_cutoff 3 -min_pair_cov 4 -paired_cutoff 0.1* and with or without *-scaffolding*. This assembly generated 84,511 contigs. The scaffolding had no effect. Since oases 0.1.x was known to generate a number of exactly identical sequences (duplicates), a reassembly was performed using TIGR assembler [48] based on the default parameters in the original *run_TA* script (ftp://ftp.jcvi.org/pub/software/assembler/), which reduced the number of contigs to 72,924. The remaining exact duplicates and reverse complement exact duplicates were removed using *prinseq-lite.pl* (http://prinseq.sourceforge.net; parameter -derep 14), yielding a total of 72,890 transcript contigs.

Functional annotation of this reference transcriptome was performed using Blast2GO version 2.4.2 [49], which enabled a semi-automated functional annotation of all contigs using a set of similarity search tools. The default parameters was used and included: i) an initial annotation with BLASTX (against the nonredundant NCBI database; e-value at 1×10^–3^); ii) a protein domain search using InterProscan; iii) an enzyme annotation using the Kyoto Encyclopedia of Genes and Genomes (KEGG); and iv) assignment of a Gene Ontology term (GO; http://www.geneontology.org/).

The taxonomic assignment of contigs (sequences from the coral host or zooxanthellae symbiont) were predicted using 3 approaches: i) top hit species from the BLASTX results (the species with the best/first sequence alignment for a given BLASTX result); ii) the percentage of GC bases, GC content of corals is approximately 42%, and 54% for zooxanthellae [50]; and iii) species specific sequence signatures based on CLaMS analysis [51].

### Differential gene expression analysis

To assess changes in gene expression regulated by low pH, SSRs from both low pH and control libraries were mapped against the reference transcriptome and counted using BWA [52]. Because RNAseq data are functions of both the molar concentration and the transcript length, the results of the mapping step were corrected and expressed as reads per kilobase per million mapped reads (RPKM; [53]). This enabled comparison of SSRs counts between transcripts and from samples derived from differing conditions [53]. To identify significantly different gene expression among conditions, the MARS method (MA plot-based methods using a random sampling model) of the R package in DEGseq was used [54]. Differences in transcripts levels between low pH and control conditions were considered statistically significant at *p*<0.0001.

### q-RT-PCR

Quantitative real-time PCR (q-RT-PCR) was used to validate expression profiles obtained from the MARS DEGseq analysis of the RNAseq data [27]. It was also used to measure expression levels of selected 48 candidate genes in *P. damicornis* subjected to complementary pH conditions (7.8 and 7.2). Total RNA was extracted, treated with DNase, and the poly(A) RNA was purified as described above. Approximately 50 ng of purified poly(A) RNA were reverse transcribed with hexamer random primers using ReverTAid H Minus Reverse Transcriptase (Fermentas). The q-RT-PCR experiments were performed on cDNAs obtained from 3 nubbins per treatments as described previously [40]. For each candidate gene the level of transcription was normalized using the mean geometric transcription rate of 3 reference sequences encoding ribosomal protein genes from *P. damicornis* (60S ribosomal protein L22, GenBank accession numbers HO112261, 60S ribosomal protein L40A, HO112283 and 60S acidic ribosomal phosphoprotein P0, HO112666). These housekeeping genes had <10% variation in expression between control and low pH conditions and were previously shown to be stable in *P. damicornis* subjected to different environmental conditions [40]. The primers used for amplification are provided in File S1.

## Results

### Assembly of the reference transcriptome

To quantify changes in gene expression between the pH 7.4 treatment and the control, we assembled a reference transcriptome *de novo*. To maximize the diversity of transcripts, the cDNA samples derived from corals exposed to various environmental conditions (pH 7.4 and its dedicated control, thermal stress, bacterial challenge, bacterial infection and a dedicated control for these last conditions) were sequenced and assembled together. After the assemble work flow, a total of 72,890 transcript contigs were generated. The functional annotation performed using Blast2GO produced the following result: i) 22,419 contigs had significant similarities to proteins of known function; ii) GO term was assigned to 23,170 contigs; iii) KEGG enzymatic codes were found for 2,837 contigs; and vi) 46,295 conserved protein domains were detected by InterProScan. Using a combination of BlastX top hit species results, GC% calculation of each contig (perl script) and sequence signature analysis (CLaMS program; [51], 27.7% and 69.8% of the contigs were predicted to belong to the symbiont and the host transcriptome, respectively. The remaining contigs could not be taxonomically attributed. The assembled and annotated reference transcriptome is publicly available at http://2ei.univ-perp.fr/telechargement/transcriptomes/blast2go_fasta_Pdamv2.zip. The raw data (untreated SSRs) for the pH treatment and its corresponding control are publicly available at http://www.ncbi.nlm.nih.gov/sra (study accession number SRP011059.1).

### Expression in 16% of the genes changes after exposure to low pH

The RNAseq approach resulted in the sequencing of 25.2 and 22.3 million Short Sequence Reads (SSRs) for the pH 8.1 and the pH 7.4 conditions, respectively. A total of approximately 10 and 9 million SSRs (∼40%) passed the quality filter and were successfully mapped on the reference transcriptome for the control and the low pH conditions, respectively. Nearly 80% (57,259 contigs) of the reference transcripts were mapped by at least one SSR from either 1 of the 2 experimental conditions.

**Figure 1:**
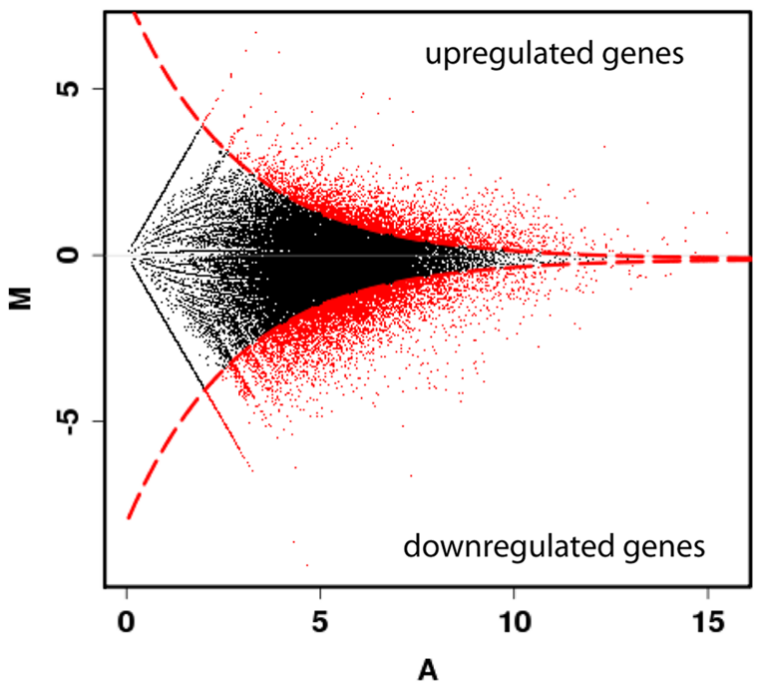
Differential gene expression between control samples and samples exposed to pH 7.4 for three weeks. The detection of genes differentially expressed in the two conditions was performed using the DEGseq R package and plotted as a MA plot. The M axis is the log2 fold change for the pH-treated sample compared with the control sample (log2 fold change  =  log2 [RPKM acid stress/RPKM control]), and the A axis is the average log2 normalized counts in both samples. Each point represents a single contig of the reference transcriptome. The red points correspond to contigs significantly differentially represented between the two conditions (*p*<0.0001). Among the 57,259 contigs analyzed, 5758 were significantly downregulated (10%) and 3204 (6%) were upregulated.

To highlight the genes significantly up or downregulated by the pH 7.4 treatment, the mapped results (RPKM) were analyzed using the MARS method in the DEGseq R package. In response to pH 7.4, 6.0% (3,204) of the transcripts were significantly upregulated and 10.0% (5,758) were downregulated (*p*<0.0001; Figure 1). Among the upregulated genes, 60.4% showed no significant similarity to known proteins, 2.7% showed significant similarity to proteins of unknown function, and 36.9% showed significant similarity to proteins of known function (Significant similarity: *e*value <1×10^–3^). Among the downregulated genes, 69.5% of their products showed no significant similarity to known proteins, 3.5% showed significant similarity to proteins of unknown function, and 27.0% showed significant similarity to proteins of known function.

**Figure 2:**
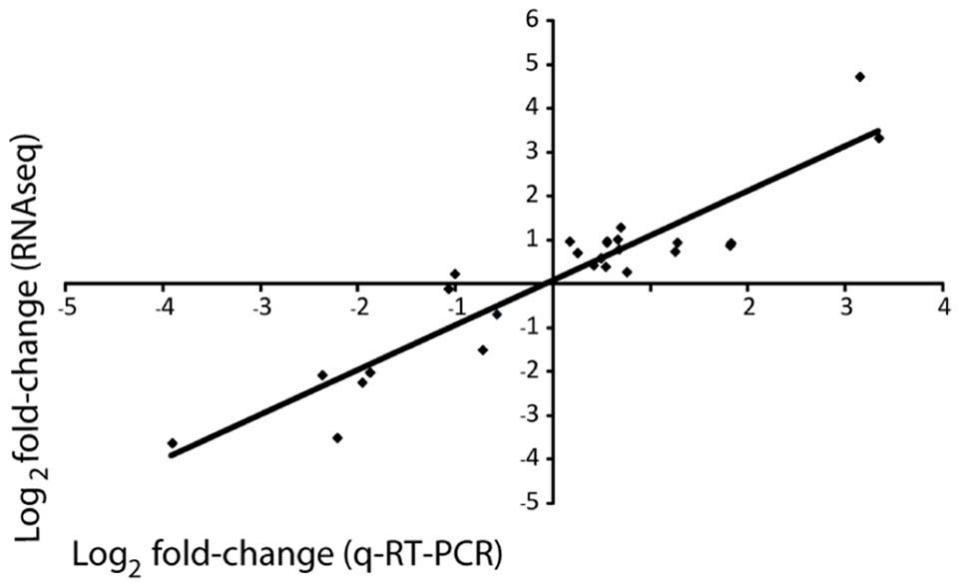
Validation of the RNAseq approach using q-RT-PCR. Twenty-five genes that whose expression was significantly different in the control and treatment conditions were arbitrary selected from highly upregulated to highly downregulated contig. Their levels of expression were quantified by q-RT-PCR, and the results were compared with those obtained using the RNAseq approach. The log2 change in expression of q-RT-PCR and RNAseq was closely correlated (*r* ^2^ = 0.86; *p*<0.0001), indicating the accuracy of the RNAseq approach for quantification.

To assess the accuracy of the quantification analysis a validation step involving an alternative method of quantification (q-RT-PCR) was conducted on 25 transcripts arbitrarily selected along the gradient of expression (from highly upregulated to highly downregulated gene) obtained from the RNAseq data. There was a significant correlation (*r* ^2^ = 0.86; *p*<0.0001; Figure 2) between the log_2_ fold change in expression (relative to the control) of the RNAseq and the q-RT-PCR data which confirmed the accuracy of the results obtained with the RNAseq data.

### Specific biological processes are altered after exposure to pH 7.4 for three weeks

**Figure 3:**
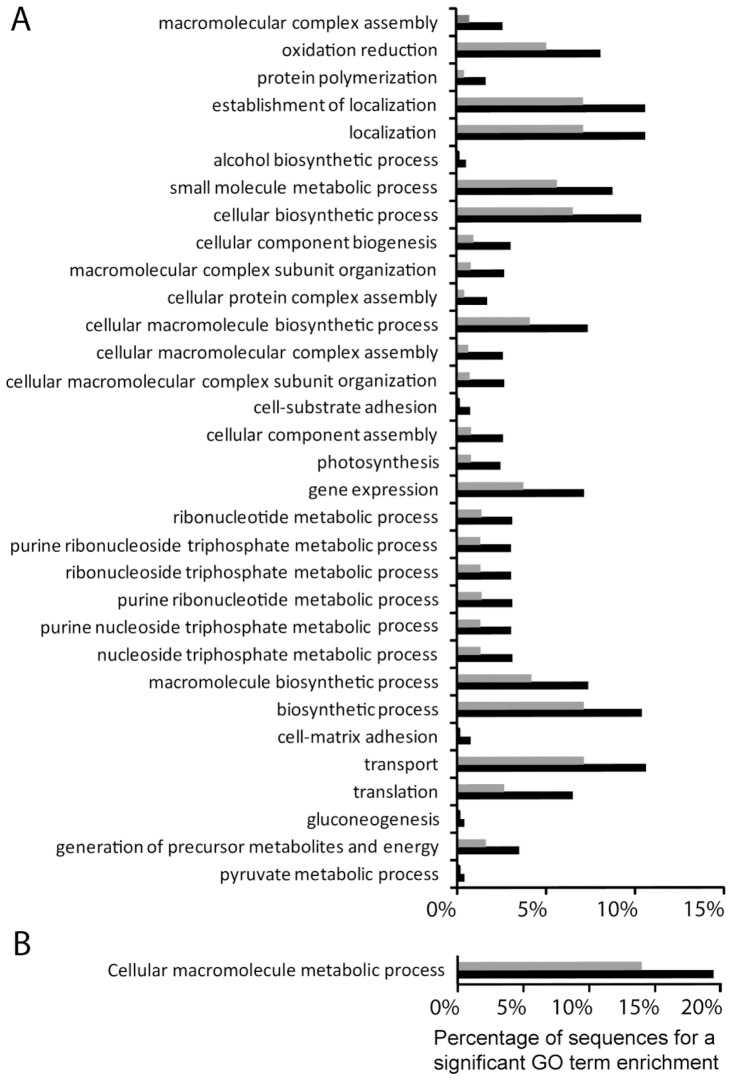
Biological functions involved in the response to pH 7.4 treatment, GO term enrichment analysis. The enrichment analysis (x axis) is expressed as the percentage of sequences at both, test (black bars) and reference (grey bars) set, for GO terms having a *p*value A) Enrichment analysis performed with the set of induced genes (“test set”, state condition, black bars) compared with genes detected in the RNAseq experiment (“reference set”, grey bars). B) Enrichment analysis performed with the set of repressed genes (“test set”, black bars) compared with all genes detected in the RNAseq experiment (reference set, grey bars). Black bars represent the percentage of induced gene (A) or repressed gene (B) in the test set, for a given GO terms. The grey bars represent the percentage of gene for a given GO terms in the reference set. The statistical test was considered significant at the 1% error level.

To study the transcriptomic changes resulting from exposure to pH 7.4 for 3 weeks, a GO term enrichment analysis (Figure 3) was performed [55]. It showed that 32 biological process categories were enriched in the subset of sequences associated with significant upregulation (Fig. 3A). Several of the functions involved were of particular interest in the context of this study, including transport, photosynthesis, gluconeogenesis, generation of precursor metabolites and energy and pyruvate metabolic process. Analysis of the subset of transcripts of significantly downregulated genes showed that only those for the GO term ‘cellular macromolecule metabolic process’ were represented at a higher rate than expected by chance (*p*<0.01; Fig. 3B). The GO terms ‘post-translational protein modification’ and ‘cellular protein metabolism’ were more significantly represented (*p*<0.05) among the downregulated set than in the reference sequence set. The numbers of genes belonging to each biological process are indicated in File S2.

### Response of some biological functions to an exposure to pH 7.8, 7.4 or 7.2 for 3 weeks

**Figure 4:**
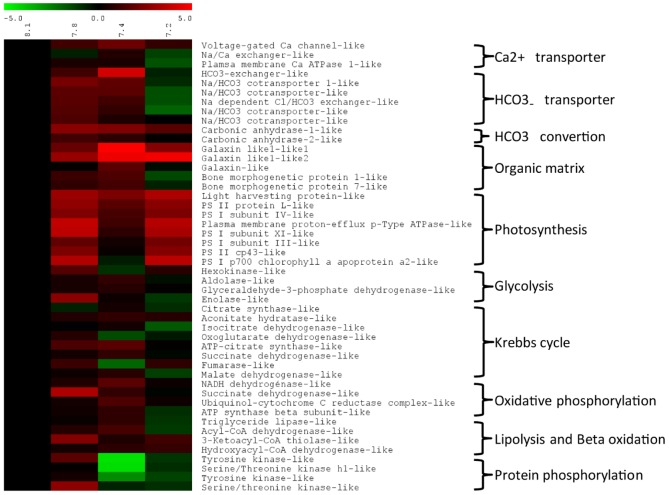
Gene expression for key biological functions following exposure to various pH levels for three weeks. The data included q-RT-PCR results for samples exposed to pH 7.8 and 7.2, and RNAseq results for samples exposed to pH 7.4. Quantification was normalized by comparison with results for exposure to pH 8.1 (present seawater pH); the results are presented as a log2 fold change in expression.

Based on GO term enrichment analysis and DEGseq results (Figure 3), 48 candidate genes significantly regulated (*p*<0.0001) were identified as corresponding to biological functions of interest in the context of our study and were selected (see File S1) and investigated further. Their transcript abundance was measured on corals exposed for 3 weeks at pH 8.1, 7.8, 7.4 and 7.2. This transcript abundance was obtained by q-RT-PCR for the pH 7.8 and 7.2 whereas the RNAseq results were used for the pH 7.4 treatment. All changes in transcript abundance were expressed relative to the control (pH 8.1). All these results are presented in Figure 4 and the numerical values are detailed in File S3.

Briefly, the GO term enrichment analysis (Figure 3) showed a significant increase in gene expression involved in the biological processes ‘transport’, which includes transport of ions and proteins. As calcification involves Ca^2+^ and inorganic carbon [56] we investigated genes encoding proteins involved in the transcellular transport of Ca^2+^ and HCO_3_^−^ ions, and carbonic anhydrases involved in converting CO_2_ into HCO_3_^−^. Among these candidates: i) The six HCO_3_^−^ transporters were upregulated at pH 7.8 and 7.4 (between 17.75 and 1.52 fold increase) and were downregulated at pH 7.2 (between 3.81 and 1.57 fold decrease); ii) a Ca^2+^ plasma membrane ATPase was upregulated at pH 7.8 and 7.4 (1.47 and 1.43 fold increase, respectively), but was downregulated at pH 7.2 (3.01 fold decrease); iii) an extracellular (upregulated at all pH between 2.51 and 3.34 fold increase) and a cytosolic carbonic anhydrase (upregulated at pH 7.8 and 7.4, 2.51 and 1.85 fold increase respectively). The Ca^2+^ plasma membrane ATPase and the two carbonic anhydrase present significant similarities with proteins that were previously shown to be involved in *Stylophora pistillata* calcification [57]–[59]. In addition to CaCO_3_, the skeleton of scleractinian corals contains organic matrix proteins [56], and their production was affected by low pH treatment. Indeed, seven genes encoding skeleton organic matrix proteins were identified in the present study. One of these genes regulated at pH 7.4 (3.5 fold increase) presents significant similarities to galaxin from *Galaxea fascicularis* [60], and the four other transcripts to the galaxin-like (only 2 of them were subjected to q-RT-PCR experiment, and were upregulated at all pH between 70.03 and 4.11 fold increase ) proteins family from *Acropora millepora* [61]. The remaining two genes had significant similarities to the bone morphogenetic protein superfamily [62] and were upregulated at pH 7.8 and 7.4 (between 2.91 and 1.93 fold increase), but downregulated at pH 7.2 (between 2.68 and 1.56 fold decrease). For a complete description of candidate genes expression see Figure 4 and File S3.

Biological functions involved in energy production (photosynthesis, glycolysis, Krebbs cycle, oxidative phosphorylation, lipolysis and beta oxidation; Figure 4) were also significantly enriched in the set of up-regulated genes (GO term enrichment analysis, Figure 3). As calcification represents approximately 13–30% of the daily coral energy demand [1], we also investigated the regulation of these metabolic related genes by q-RT-PCR for the 3 pH treatments. Among the eight candidates genes involved in photosynthesis, seven were upregulated (between 1.15 and 5.54 fold increase) and one, the PS I p700 chlorophyll a apoprotein a2-like was downregulated at pH 7.4 (1.96 fold decrease). Three of the four candidate genes involved in glycolysis were upregulated at pH 7.8 and 7.4 (between 6.68 and 1.22 fold increase) and two were downregulated at pH 7.2 (between 2.11 and 1.22 fold decrease). Among the eight the candidates genes involved in the Krebs cycle six shows upregulations at pH 7.8 and/or 7.4 (between 3.36 and 1.33 fold increase) and five were downregulated at pH 7.2 (between 2.36 to 1.1 fold decrease). Four of the genes involved in oxidative phosphorylation were upregulated at pH 7.8 and 7.4 (between 10.85 and 1.28 fold increase), but one become downregulated at pH 7.2 (1.89 fold decrease). One of the genes involved in lipolysis was upregulated at pH 7.8 and 7.4 (1.15 and 1.95 fold increase respectively) but was downregulated by a factor of 2 in the pH 7.2 treatment. Finally, the three candidate genes involved in beta oxidation were upregulated at all pH (between 5.98 and 1.37 fold increase), except the Acyl-CoA dehydrogenase-like that was downregulated by a factor of 2.17 at pH 7.2. For a complete description of candidate genes expression see the Figure 4 and File S3.

Finally, very few biological processes were significantly enriched in the genes downregulated at pH 7.4 (GO term enrichment analysis, Figure 3). However, these included the category ‘post-translational protein modification’ (GO term enrichment analysis; *p*<0.05), which contains genes encoding kinases. Because of their important role in signal transduction, four of those were selected, two encoded tyrosine protein kinase while the other two genes encoded serine/threonine protein kinases. These four genes were downregulated at pH 7.4 and 7.2 (between 22.01 and 1.82 fold decrease). For a complete description of candidate genes expression see the Figure 4 and File S3.

## Discussion

### Validation of the RNAseq approach to the study of coral responses to low pH

An RNAseq approach was used to compare the transcriptomes of corals maintained under seawater pH conditions with those of corals exposed to pH 7.4 (an extreme value of today or near future natural variability) for a 3-weeks period. Based on our reference transcriptome, the constant expression of 84% of the genes evidenced that *Pocillopora damicornis* transcriptome is not completely disturbed by the treatment and that its response to pH 7.4 is mediated by the modification of 16% of its transcriptome. Complementary analysis of these 16% of genes allowed us to identity several molecular pathways and candidate genes putatively involved in the response to low pH. In order to better understand the response of these affected biological functions at a pH 7.8 and 7.2, we measured the transcript abundance of genes belonging to these affected functions by q-RT-PCR method.

The quantification of gene expression by RNAseq requires a reference genome/transcriptome for the mapping step [53]. However, such molecular data are scarce, especially for non-model species. This explains why few global transcriptomic analyses have been undertaken using non-model species. Among scleractinian corals such analyses were only possible for a few species such as *Acropora millepora* and *A. digitifera* because their transcriptome or genome data were available [27], [63], [64]. Recent progress in the development of next generation sequencing made it possible to deal with this problem. In a two-step process, the sequences obtained under different physiological conditions were first used to assemble the transcriptome. Secondly, the sequences for each condition were used for quantification of gene expression. The use of transcripts from different environmental conditions allowed us also to cover the largest possible range of mRNAs resulting from differential transcription and alternative splicing [65], [66]. Comparison of the quantitative data obtained using the RNAseq and the q-RT-PCR approaches (the latter performed on 25 arbitrarily selected genes) showed a significant correlation between these two methods of quantification. The correlation obtained (*r* ^2^ = 0.86) is comparable to those reported in other studies by Oliver and collaborators [67], *r* ^2^ = 0.83; Wang and collaborators [68], *r* ^2^ = 0.83; Castruita and collaborators [69], *r* ^2^ = 0.95; and Meyer and collaborators [27] *r* ^2^ = 0.74). This confirms that the two-step RNAseq approach we used is suitable for measuring gene expression in corals exposed to various environmental conditions.

### Specific upregulation of biological functions under low pH

The overall pattern of gene expression showed that 6% of the genes were significantly upregulated during exposure to pH 7.4 compared to pH 8.1. One important finding is that genes coding for organic matrix proteins and proteins involved in the transport of Ca^2+^ and HCO_3_^-^ are modulated under low pH conditions, suggesting that low pH affects the calcification process at the transcriptomic level. We showed that genes coding for HCO_3_^−^ and Ca^2+^ transporters and carbonic anhydrases were upregulated at low pH values (7.8 and 7.4) but downregulated at the extreme level of pH 7.2 (Figure 4). A similar effect was observed for genes encoding skeletal organic matrix proteins (similar to BMP1 and BMP7 of the bone morphogenic protein superfamily, and the galaxin superfamily).

These results contrasts with some of those obtained in two recent works: one studying by RNAseq the short term (3 days exposure) response of *Acropora millepora* primary polyps to pH 7.96 and 7.86 compared to pH 8.16 [23], and the second one studying by microarray the short and midterm (1 day and 28 days) responses of adults *A. millepora* to pH 7.85 and 7.65 compared to pH 8.1 [17]. Among these three studies, if the two using *A. millepora* as a model species are the most realistic in terms of pH exposure (pH∼7.9), ours offer the opportunity to study the response to pH below 7.6 (7.4 and 7.2), which can exacerbate the transcriptomic response. Concerning the comparison with the study of Moya and collaborators [23], in condition where pH between studies is the closest (7.8), their results are in agreement with ours for the genes coding for Ca^2+^ transporters and Galaxin-like protein. However, carbonic anhydrase and some organic matrix encoding genes are downregulated in their experiment while it is the contrary in ours. These contradictions are not surprising since we worked at a different time-scale, on a different species and on adult corals whereas they worked on early aposymbiotic life stages. However, even when considering the same species *A. millepora*, Moya and collaborators [23] have obtained results different from the ones obtained by Kaniewska and collaborators [17]. For example, the genes coding for organic matrix proteins (such as galaxin) clearly shows that effect of *p*CO_2_ is dependent on the life stage. Then differences obtained between our study and the study of Kaniewska and collaborators [17] mainly concern the opposite pattern of expression for the metabolic related genes. Indeed, we measured upregulation while they highlight downregulation. In this last case the contradiction reported may be the results of i) phylogenetic differences (*Acropora* belongs to the “complex” coral clade whereas *Pocillopora* belongs to the “robust” coral clade; [24], [25]), ii) and/or experimental protocol differences (light and nutrition were different in the two studies) iii) and/or differences in previous life history (corals are from different geographical origin). Such differences in the response of marine invertebrates belonging to the same phyla were also previously described. In the sea urchin group, *Paracentrotus lividus* and *Strongylocentrotus purpuratus* larvae showed mixed transcriptomic responses for ortholog genes when larvae are submitted to a comparable exposition to low pH. In *S.purpuratus* the genes involved in calcification processes msp130 and SM30 were down-regulated at pH 7.7 [70], while the same gene in *P. lividus* were not at pH 7.7, but up-regulated at pH 7.0 [71]. In coccolithophores, a decreasing calcification between ancient (past 400 years) and actual specimens was shown [72]. However they also discovered a heavily calcified coccolithophore in modern water with low pH, *Emiliana* huxleyi morphotype evidencing variation in responses of phyla to environmental forcing factors [72]. In the case of marine mollusks, low pH induced mainly a decrease of calcification both at the transcriptomic and the animal level, but some exceptions were reported [73]–[77]. These data highlight the difficulties to extrapolate the results obtained from one species to the whole phylum.

**Figure 5:**
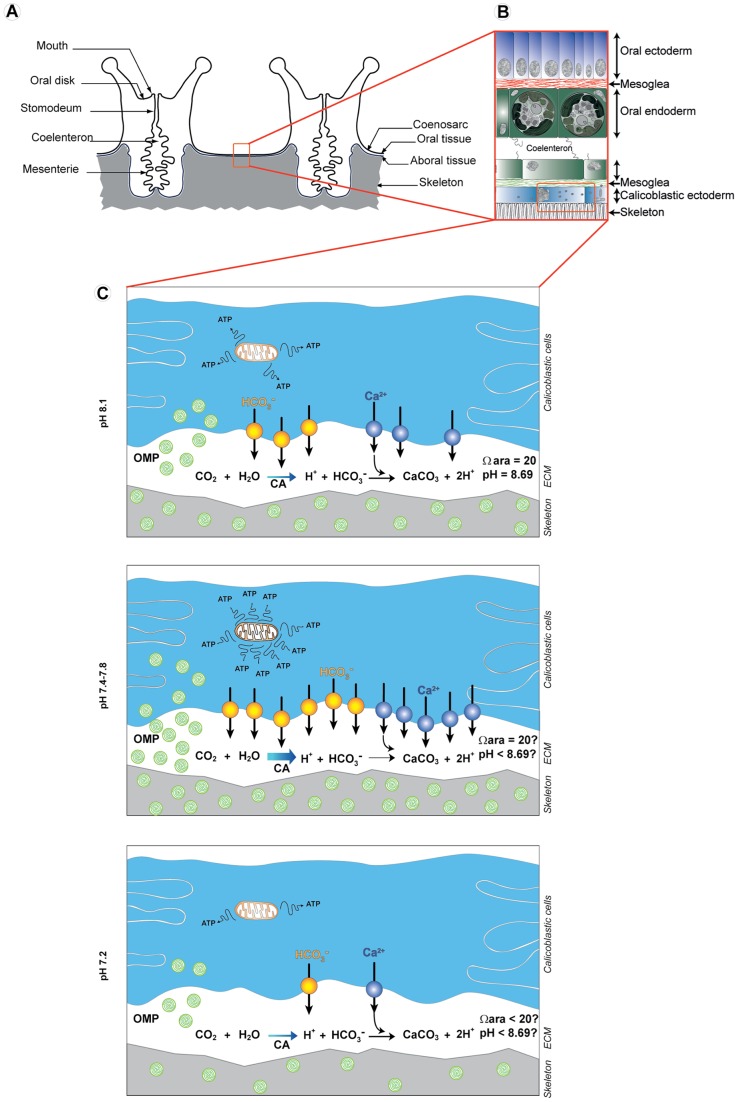
Schematic representation of key biological processes regulated under different levels of low pH. A) Anatomy of two polyps of a coral colony. B) Representation of the coenosarc composed of the oral and aboral tissues. C) Magnification of the calicoblastic ectoderm showing the calicoblastic cells with the up and downregulation of the key biological processes at different pH. At pH 7.8 or 7.4, Ca^2+^ and HCO_3_^−^ transport to the extracellular calcifying medium (ECM) increase through an increasing number of Ca^2+^ and HCO_3_^−^ transporters. The rate of CO_2_ conversion to HCO_3_^−^ increases through an upregulation of carbonic anhydrase (CA). Production and secretion of organic matrix protein (OMP round green shape) also increase. This process may help to maintain a sufficient aragonite saturation state (Ω_arag_) despite a decrease in pH, enabling to maintain the same calcification in less favorable pH conditions. This process is energetically costly and need an increase in energy production (ATP) through an increasing metabolic activity and a general trade off mechanism. Finally, at a pH 7.2, the coral cannot cope anymore with such a low pH and there could be a physiological collapse of the colony. Values of omega aragonite (Ω) and pH in the ECM at pH 8.1 are from [97].

In our study, we hypothesize that the upregulation of genes encoding proteins involved in ion transport counteract the negative effect of low pH on the calcification process (Figure 5). If the aragonite saturation state (Ω_arag_) decreased in the extracellular calcifying medium (ECM) due to a decreasing pH in the ECM [78], [79], the upregulation of ion transporters would allow to increase ion concentration and to maintain the same calcification efficiency in a less favorable pH conditions (Figure 5). Upregulation of genes involved in ion transport could indeed help to increase the saturation state of aragonite, which depends on the concentration of Ca^2+^ and CO_3_^2−^ (Figure 5). The increase in expression of genes coding for organic matrix proteins needs to be further investigated. However, we postulate that they may modify nucleation, crystal growth inhibition and orientation, and because of their negative charge help to locally increase Ω_arag_ by trapping Ca^2+^ ions [1], [56]. It is also possible that the relative composition of the skeleton (inorganic versus organic matrix) could change under low pH; an increase in the organic matrix proportion could help sustain coral growth (Figure 5). In summary, the concomitant and putative additive effect of the increase in ion transport and deposition of organic matrix proteins may help *P. damicornis* to cope with low pH exposure (Figure 5). This hypothesis is supported by recent studies performed on marine calcifying invertebrates at several levels of organization. In larvae of the sea urchin *P. lividus*, there was no effect of low pH on the calcification rate at the organism level with an upregulation of biomineralization related genes at the transcriptomic level [71]. In the mollusk *Mytilus edulis* exposed to low pH, gene transcription of a tyrosinase putatively involved in the periostracum formation was strongly upregulated and organic matrix protein coding gene remained expressed at a high level. Both mechanisms were associated to the protection and the maintenance of shell formation [74]. In the red abalone *Haliothis rufescens*, organic matrix protein coding genes remained expressed at a high level during exposure to low pH [80]. At the organism level examples of calcification increase were reported in several organisms such as *Crepidula fornicata*, *Arbacia punctulata* [77]. Finally, a recent work on the coral *Stylophora pistillata* has shown that between pH 8.1 and pH 7.4, the animal controls sufficiently the pH in the ECM to allow the maintenance of calcification [81]. Even if all these examples strengthen our hypothesis, it should be confirmed in *P. damicornis* further by direct quantification of skeletal Ca_2_^+^ incorporation, organic matrix skeletal content, and measurements of pH in the ECM.

In tropical corals energy is supplied by prey capture (heterotrophic nutrition) and translocation of photosynthates from symbionts to their host [82]. The upregulation of genes involved in heterotrophic and autotrophic pathways may reflect an increase in coral energetic requirements under low pH stress. In this context, it is known that calcification is an energetically costly process consuming up to 30% of the daily energy budget [1]; the need to maintain calcification under stress conditions could in part explain the increase in energetic needs and energy production (Figure 5). In this context, the upregulation of lipolysis and beta-oxidation metabolic pathways shows that corals may use their fatty acid reserves under low pH conditions. However, these reserves are not inexhaustible and should be replenished, whereas global climate change is predicted to cause a decrease in ocean primary production [5], [83], [84]. Thus, it is uncertain whether corals can maintain their energy production from heterotrophic and autotrophic resources when primary production is predicted to decrease.

Usually high CO_2_ concentration driving seawater pH decrease is known to induce bleaching of photosymbiotic organisms such as reef-building coral and foraminifera [17], [22], [85], even if the photophysiology of the phototrophic symbiont shows mixed responses to this treatment (see introduction section). Our study is the first to show the upregulation of several photosynthetic related genes at pH<8.1. The lack of photophysiological data in the present study makes it difficult to interpret such upregulations but argue in favor of an increasing photosynthetic capability of the holobiont *P. damicornis* under pH of 7.8, 7.4 and 7.2. Such a positive effect of high CO_2_ concentration on photosymbiotic species just begins to be revealed in the literature. Indeed, the acoel worm *Symsagittifera roscoffensis* entering in symbiosis with the microalgae *Tetraselmis convolutae* shows a remarkable symbiosis stability even at a very low pH level (up to pH 6) [86]. In a second example, the sea anemone *Anthopleura elegantissima*/*Symbiodinium muscatinei* photosymbiosis, high *p*CO_2_ level (pH 8.08 and pH 7.35) induces a higher rate of photosynthesis and mitotic index of the algae compared to pH 8.1 [87]. Under such conditions the cnidarian host received more of their respiratory carbon from the symbiont that under the actual pH condition [87]. These results taking together confirmed the hypothesis that photosymbiosis could be resistant to high *p*CO_2_, and that the negative effect of this high *p*CO_2_ could be the results of indirect impact at other levels (hypothesis proposed in [86]).

### Uncoordinated downregulation of biological functions under low pH

During exposure to pH 7.4 compared to 8.1, 10% of genes were downregulated and 6% were upregulated. Although more genes were downregulated, the enrichment analysis shows that the upregulation of genes was more readily explained and informative. A total of 32 biological functions were upregulated under low pH conditions (*p*>0.01), while only 1, the ‘cellular macromolecule metabolic process’ was downregulated (Fig. 3; *p*>0.01) despite a comparable number of contigs with a Gene Ontology term (1696 for the downregulated set, 1495 for the upregulated set). Such a results may reflect that the downregulation measured are un or less coordinated than the upregulation. This kind of uncoordinated downregulation of genes has been reported in several other animal species exposed to stress [88]–[90]. A similar response was also observed in the coral *Montastraea faveolata* subject to thermal stress [91]. Downregulation could reflect a trade-off mechanism induced by the low pH treatment, providing energy savings necessary for efficient stress responses (Figure 5). This hypothesis is supported by the results of the q-RT-PCR experiments performed on selected host genes. Indeed, the level of downregulation increased concomitantly with the pH level. At pH 7.2 the downregulation was maximal and, as it has been observed and hypothesized in other species subjected to extreme physiological stress conditions, this may reflect a trade-off mechanism [89], [91]–[94].

Post-translational protein modification was one of the biological functions downregulated (GO term enrichment analysis, *p*<0.05) during low pH treatment, as evidenced by the substantial downregulation of several genes encoding protein kinases (serine/threonine kinase and tyrosine kinase). Protein kinases phosphorylate proteins to modulate their activity, and play key roles in signal transduction pathways [95]. The four protein kinases selected for q-RT-PCR analysis showed similar patterns of expression, with upregulation occurring at pH 7.8 and downregulation occurring at pH 7.4 and 7.2. This variable pattern of transcription could reflect the disturbance or molecular plasticity of signaling in coral cells under stress. Such results are strengthened by a proteomic study of the barnacle *Balanus amphitrite* exposed to low pH. Indeed, under low pH condition the phosphoproteome is modified revealing a regulation of kinase activity and/or synthesis [96]. The biological effects of all these downregulation events were difficult to resolve because of the very large number of pathways in which kinases are involved, and this will require further study.

## Supporting Information

File S1 **Biological function, annotation, BlastX top hit species, BlastX evalue, contig number and primer sequence of the selected candidate genes.**
(DOCX)

File S2 **Table results of the GO term enrichment analysis.**
(DOCX)

File S3 **Numerical value corresponding to the qRT-PCR.** Quantification was normalized by comparison with results for exposure to pH 8.1 (present seawater pH); the results are presented as a log2 fold change in expression.
(DOCX)

## References

[1] Allemand D, Tambutté E, Zoccola D, Tambutté S (2011) Coral calcification, cells to reefs. In: Dubinsky Z, Stambler N, editors. Coral reefs: an ecosystem in transition. New York: Springer. 119–150.

[2] Wilkinson C (2008) Status of coral reefs of the world. In: Wilkinson C, editor. Status of Coral Reefs of the World. Townsville: Global Coral Reef Monitoring Network and Reef and Rainforest Research Center. 296.

[3] Bellwood DR, Hughes TP, Folke C, Nyström M (2004) Confronting the coral reef crisis. Nature 429: 827–833. 15215854 10.1038/nature02691

[4] De'ath G, Lough JM, Fabricius KE (2009) Declining coral calcification on the Great Barrier reef. Science 323(5910): 116–119. 19119230 10.1126/science.1165283

[5] Hoegh-Guldberg O, Bruno JF (2010) The impact of climate change on the world's marine ecosystems. Science 328(5985): 1523–1528. 20558709 10.1126/science.1189930

[6] Albright R, Mason B, Miller M, Langdon C (2011) Ocean acidification compromises recruitment success of the threatened Caribbean coral *Acropora palmata* . Proc Natl Acad Sci USA 107(47): 20400. 10.1073/pnas.1007273107 PMC2996699 21059900

[7] Cohen AL, McCorkle DC, de Putron S, Gaetani GA, Rose KA (2009) Morphological and compositional changes in the skeletons of new coral recruits reared in acidified seawater: Insights into the biomineralization response to ocean acidification. Geochem Geophys Geosyst 10(7).

[8] Erez J, Reynaud S, Silverman J, Schneider K, Allemand D (2011) Coral calcification under ocean acidification and global change. In: Dubinsky Z, Stambler N, editors. Coral Reefs: An Ecosystem in Transition. New York: Springer. 151–176.

[9] Iguchi A, Ozaki S, Nakamura T, Inoue M, Tanaka Y, et al (2012) Effects of acidified seawater on coral calcification and symbiotic algae on the massive coral *Porites australiensis* . Mar Environ Res 73: 32–36. 22115919 10.1016/j.marenvres.2011.10.008

[10] Inoue M, Suwa R, Suzuki A, Sakai K, Kawahata H (2011) Effects of seawater pH on growth and skeletal U/Ca ratios of *Acropora digitifera* coral polyps. Geophysical Research Letters 38(12): L12809.

[11] Krief S, Hendy EJ, Fine M, Yam R, Meibom A, et al (2010) Physiological and isotopic responses of scleractinian corals to ocean acidification. Geochim Cosmochim Acta 74(17): 4988–5001.

[12] Langdon C, Atkinson MJ (2005) Effect of elevated *p*CO_2_ on photosynthesis and calcification of corals and interactions with seasonal change in temperature/irradiance and nutrient enrichment. J Geophys Res 110(C9): C09S07.

[13] Leclercq N, Gattuso JP, Jaubert J (2002) Primary production, respiration, and calcification of a coral reef mesocosm under increased CO_2_ partial pressure. Limnol Oceanogr 47(2): 558–564.

[14] Marubini F, Ferrier-Pages C, Furla P, Allemand D (2008) Coral calcification responds to seawater acidification: a working hypothesis towards a physiological mechanism. Coral Reefs 27(3): 491–499.

[15] Ries JB, Cohen AL, McCorkle DC (2009) Marine calcifiers exhibit mixed responses to CO2-induced ocean acidification. Geology 37(12): 1131.

[16] Houlbrèque F, Rodolfo-Metalpa R, Jeffree R, Oberhänsli F, Teyssiè JL, et al (2012) Effects of increased *p*CO_2_ on zinc uptake and calcification in the tropical coral *Stylophora pistillata* . Coral Reefs 31(1): 1–9.

[17] Kaniewska P, Campbell PR, Kline DI, Rodriguez-Lanetty M, Miller DJ, et al (2012) Major cellular and physiological impacts of ocean acidification on a reef-building coral. PLoS ONE 7(4): e34659. 22509341 10.1371/journal.pone.0034659 PMC3324498

[18] Reynaud S, Leclercq N, Romaine-Lioud S, Ferrier-Pagès C, Jaubert J, et al (2003) Interacting effects of CO_2_ partial pressure and temperature on photosynthesis and calcification in a scleractinian coral. Glob chang biol 9(11): 1660–1668.

[19] Fabricius KE, Langdon C, Uthicke S, Humphrey C, Noonan S, et al (2011) Losers and winners in coral reefs acclimatized to elevated carbon dioxide concentrations. Nat Clim Chang 1(3): 165–169.

[20] Rodolfo-Metalpa R, Houlbrèque F, Tambuttè E, Boisson F, Baggini C, et al (2011) Coral and mollusc resistance to ocean acidification adversely affected by warming. Nat Clim Chang 1(6): 308–312.

[21] Fine M, Tchernov D (2007) Scleractinian coral species survive and recover from decalcification. Science 315(5820): 1811. 17395821 10.1126/science.1137094

[22] Anthony KRN, Kline DI, Diaz-Pulido G, Dove S, Hoegh-Guldberg O (2008) Ocean acidification causes bleaching and productivity loss in coral reef builders. Proc Natl Acad Sci USA 105(45): 17442–17446. 18988740 10.1073/pnas.0804478105 PMC2580748

[23] Moya A, Huisman L, Ball EE, Hayward DC, Grasso LC, et al (2012) Whole transcriptome analysis of the coral *Acropora millepora* reveals complex responses to CO_2_-driven acidification during the initiation of calcification. Mol Ecol 21(10): 2440–2454. 22490231 10.1111/j.1365-294X.2012.05554.x

[24] Kitahara MV, Cairns SD, Stolarski J, Blair D, Miller DJ (2010) A comprehensive phylogenetic analysis of the Scleractinia (Cnidaria, Anthozoa) based on mitochondrial CO1 sequence data. PloS ONE 5(7): e11490. 20628613 10.1371/journal.pone.0011490 PMC2900217

[25] Romano SL, Cairns SD (2000) Molecular phylogenetic hypotheses for the evolution of scleractinian corals. Bull Mar Sci 67(3): 1043–1068.

[26] Franssen SU, Gu J, Bergmann N, Winters G, Klostermeier UC, et al (2011) Transcriptomic resilience to global warming in the seagrass *Zostera marina*, a marine foundation species. Proc Natl Acad Sci USA 108(48): 19276–19281. 22084086 10.1073/pnas.1107680108 PMC3228437

[27] Meyer E, Aglyamova GV, Matz MV (2011) Profiling gene expression responses of coral larvae (*Acropora millepora*) to elevated temperature and settlement inducers using a novel RNA-Seq procedure. Mol Ecol 20(17): 3599–3616. 21801258 10.1111/j.1365-294X.2011.05205.x

[28] Runcie DE, Garfield DA, Babbitt CC, Wygoda JA, Mukherjee S, et al (2012) Genetics of gene expression responses to temperature stress in a sea urchin gene network. Mol Ecol 21(18): 4547–4562. 22856327 10.1111/j.1365-294X.2012.05717.x PMC3866972

[29] Veron JEN (2000) Corals of the World; Stafford-Smith M, editor. Townsville: Australian Institute of Marine Science. 463 p.

[30] Manzello DP (2010) Ocean acidification hot spots: Spatio-temporal dynamics of the seawater C0_2_ system of eastern Pacific coral reefs. Limnol Oceanogr 55(1): 239.

[31] Ben-Haim Y, Rosenberg E (2002) A novel *Vibrio* sp. pathogen of the coral *Pocillopora damicornis* . Mar Biol 141: 47–55.

[32] Hashimoto K, Shibuno T, Murayama-Kayano E, Tanaka H, Kayano T (2004) Isolation and characterization of stress-responsive genes from the scleractinian coral *Pocillopora damicornis* . Coral Reefs 23: 485–491.

[33] Loya Y, Sakai K, Yamazato K, Nakano Y, Sambali R, et al (2001) Coral bleaching: the winners and the losers. Ecol Lett 4(2): 122–131.

[34] Gagliano M, McCormick M, Moore J, Depczynski M (2010) The basics of acidification: baseline variability of pH on Australian coral reefs. Mar Biol 157(8): 1849–1856.

[35] Santos IR, Glud RN, Maher D, Erler D, Eyre BD (2011) Diel coral reef acidification driven by porewater advection in permeable carbonate sands, Heron Island, Great Barrier Reef. Geophysical Research Letters 38(3): L03604.

[36] Dickson AG, Sabine CL, Christian JR (2007) Guide to best practices for ocean CO_2_ measurements. PICES Special Publication 3: 191.

[37] van Heuven S, Pierrot D, Lewis E, Wallace DWR (2009) MATLAB Program developed for CO_2_ system calculations.

[38] Mehrbach C, Culberson CH, Hawley JE, Pytkowicz RM (1973) Measurement of the apparent dissociation constants of carbonic acid in seawater at atmospheric pressure. Limnol Oceanogr 18: 897–907.

[39] Dickson AG, Millero FJ (1987) A comparison of the equilibrium constants for the dissociation of carbonic acid in seawater media. Deep-Sea Res 34(1733–1743).

[40] Vidal-Dupiol J, Ladrière O, Meistertzheim AL, Fouré L, Adjeroud M, et al (2011) Physiological responses of the scleractinian coral *Pocillopora damicornis* to bacterial stress from *Vibrio coralliilyticus* . J Exp Biol 214: 1533–1545. 21490261 10.1242/jeb.053165

[41] Traylor-Knowles N, Granger B, Lubinski T, Parikh J, Garamszegi S, et al (2011) Production of a reference transcriptome and a transcriptomic database (PocilloporaBase) for the cauliflower coral, *Pocillopora damicornis* . BMC Genomics 12(1): 585. 22126435 10.1186/1471-2164-12-585 PMC3339375

[42] Moya A, Tambutté S, Bertucci A, Tambutté E, Lotto S, et al (2008) Carbonic anhydrase in the scleractinian coral *Stylophora pistillata* . J Biol Chem 283(37): 25475–25484. 18617510 10.1074/jbc.M804726200

[43] Fox S, Sergei F, Mockler TC (2010) Applications of ultra-hignt-throughput sequencing. In: Belostotky DA, editor. Plant systems Biology. New York: Humana Press. 79–108.

[44] Pomraning KR, Smith KM, Bredeweg EL, Phatale PA, Connolly LR, et al.. (2012) Paired-end library preparation for rapid genome sequencing. Fungal Secondary Metabolism. New York: Humana Press.

[45] Pomraning KR, Smith KM, Freitag M (2009) Genome-wide high throughput analysis of DNA methylation in eukaryotes. Methods 47: 142–150. 18950712 10.1016/j.ymeth.2008.09.022

[46] Zerbino DR (2010) Using the Velvet de novo Assembler for Short-Read Sequencing Technologies: John Wiley & Sons, Inc. 1–12 p. 10.1002/0471250953.bi1105s31 PMC2952100 20836074

[47] Schulz MH, Zerbino DR, Vingron M, Birney E (2012) Oases: Robust de novo RNA-seq assembly across the dynamic range of expression levels. Bioinformatics. 10.1093/bioinformatics/bts094 PMC3324515 22368243

[48] Sutton GG, White O, Adams MD, Kerlavage AR (1995) TIGR Assembler: A new tool for assembling large shotgun sequencing projects. Genom Sci Tech 1(1): 9–19.

[49] Conesa A, Götz S, Garcia-Gomez JM, Terol J, Talon M, et al (2005) Blast2GO: a universal tool for annotation, visualization and analysis in functional genomics research. Bioinformatics 21(18): 3674–3676. 16081474 10.1093/bioinformatics/bti610

[50] Sabourault C, Ganot P, Deleury E, Allemand D, Furla P (2009) Comprehensive EST analysis of the symbiotic sea anemone *Anemonia viridis* . BMC Genomics 10(1): 333. 19627569 10.1186/1471-2164-10-333 PMC2727540

[51] Pati A, Heath LS, Kyrpides NC, Ivanova N (2011) ClaMS: A Classifier for Metagenomic Sequences. Stand Genom Sci 5(2): 248. 10.4056/sigs.2075298 PMC3235515 22180827

[52] Li H, Durbin R (2009) Fast and accurate short read alignment with Burrows-Wheeler transform. Bioinformatics 25(14): 1754–1760. 19451168 10.1093/bioinformatics/btp324 PMC2705234

[53] Mortazavi A, Williams BA, McCue K, Schaeffer L, Wold B (2008) Mapping and quantifying mammalian transcriptomes by RNA-Seq. Nat Methods 5(7): 621–628. 18516045 10.1038/nmeth.1226 PMC13303166

[54] Wang L, Feng Z, Wang X, Wang X, Zhang X (2010) DEGseq: an R package for identifying differentially expressed genes from RNA-seq data. Bioinformatics 26(1): 136–138. 19855105 10.1093/bioinformatics/btp612

[55] Bluthgen N, Brand K, Cajavec B, Swat M, Herzel H, et al.. (2004) Biological profiling of gene groups utilizing Gene Ontology. Arxiv preprint q-bio/0407034. 16362912

[56] Tambutté S, Holcomb M, Ferrier-Pagès C, Reynaud S, Tambutté E, et al (2011) Coral biomineralization: from the gene to the environment. J Exp Mar Biol Ecol 408(1): 58–78.

[57] Bertucci A, Tambutté S, Supuran C, Allemand D, Zoccola D (2011) A new coral carbonic anhydrase in *Stylophora pistillata* . Mar Biotechnol 13(5): 992–1002. 21318259 10.1007/s10126-011-9363-x

[58] Moya A, Ferrier-Pagès C, Furla P, Richier S, Tambutté E, et al (2008) Calcification and associated physiological parameters during a stress event in the scleractinian coral *Stylophora pistillata* . Comp Biochem Physiol A-Mol Integr Physiol 151(1): 29–36. 18606553 10.1016/j.cbpa.2008.05.009

[59] Zoccola D, Tambutte E, Kulhanek E, Puverel S, Scimeca JC, et al (2004) Molecular cloning and localization of a PMCA P-type calcium ATPase from the coral *Stylophora pistillata* . Biochim Biophys Acta 1663: 117–126. 15157614 10.1016/j.bbamem.2004.02.010

[60] Fukuda I, Ooki S, Fujita T, Murayama E, Nagasawa H, et al (2003) Molecular cloning of a cDNA encoding a soluble protein in the coral exoskeleton. Biochem Biophys Res Commun 304(1): 11–17. 12705876 10.1016/s0006-291x(03)00527-8

[61] Reyes-Bermudez A, Lin Z, Hayward D, Miller D, Ball E (2009) Differential expression of three galaxin-related genes during settlement and metamorphosis in the scleractinian coral *Acropora millepora* . BMC Evol Biol 9(1): 178. 19638240 10.1186/1471-2148-9-178 PMC2726143

[62] Zoccola D, Moya A, Béranger G, Tambutté E, Allemand D, et al (2009) Specific expression of BMP2/4 ortholog in biomineralizing tissues of corals and action on mouse BMP receptor. Mar Biotechnol 11(2): 260–269. 18795368 10.1007/s10126-008-9141-6

[63] Meyer E, Aglyamova G, Wang S, Buchanan-Carter J, Abrego D, et al (2009) Sequencing and de novo analysis of a coral larval transcriptome using 454 GSFlx. BMC Genomics 10(1): 219. 19435504 10.1186/1471-2164-10-219 PMC2689275

[64] Shinzato C, Shoguchi E, Kawashima T, Hamada M, Hisata K, et al (2011) Using the *Acropora digitifera* genome to understand coral responses to environmental change. Nature 476: 320–323. 21785439 10.1038/nature10249

[65] Robinson JL, Hall JR, Charman M, Ewart KV, Driedzic WR (2011) Molecular analysis, tissue profiles, and seasonal patterns of cytosolic and mitochondrial GPDH in freeze-resistant rainbow smelt (*Osmerus mordax*). Physiol Biochem Zool 84(4): 363–376. 21743250 10.1086/660162

[66] Zhu J, Dong C-H, Zhu J-K (2007) Interplay between cold-responsive gene regulation, metabolism and RNA processing during plant cold acclimation. Curr Opin Plant Biol 10(3): 290–295. 17468037 10.1016/j.pbi.2007.04.010

[67] Oliver H, Orsi R, Ponnala L, Keich U, Wang W, et al (2009) Deep RNA sequencing of L. monocytogenes reveals overlapping and extensive stationary phase and sigma B-dependent transcriptomes, including multiple highly transcribed noncoding RNAs. BMC Genomics 10(1): 641. 20042087 10.1186/1471-2164-10-641 PMC2813243

[68] Wang Z, Kadouri D, Wu M (2011) Genomic insights into an obligate epibiotic bacterial predator: *Micavibrio aeruginosavorus* ARL-13. BMC Genomics 12(1): 453. 21936919 10.1186/1471-2164-12-453 PMC3189940

[69] Castruita M, Casero D, Karpowicz SJ, Kropat J, Vieler A, et al (2011) Systems biology approach in chlamydomonas reveals connections between copper nutrition and multiple metabolic steps. Plant Cell Onl 23(4): 1273–1292. 10.1105/tpc.111.084400 PMC3101551 21498682

[70] Stumpp M, Dupont S, Thorndyke MC, Melzner F (2011) CO_2_ induced seawater acidification impacts sea urchin larval development II: Gene expression patterns in pluteus larvae. Comparative Biochemistry and Physiology-Part A: Molecular & Integrative Physiology 160(3): 320–330. 10.1016/j.cbpa.2011.06.023 21742049

[71] Martin S, Richier S, Pedrotti ML, Dupont S, Castejon C, et al (2011) Early development and molecular plasticity in the Mediterranean sea urchin *Paracentrotus lividus* exposed to CO_2_-driven acidification. J Exp Biol 214(8): 1357–1368. 21430213 10.1242/jeb.051169

[72] Beaufort L, Probert I, de Garidel-Thoron T, Bendif EM, Ruiz-Pino D, et al (2011) Sensitivity of coccolithophores to carbonate chemistry and ocean acidification. Nature 476(7358): 80–83. 21814280 10.1038/nature10295

[73] Dickinson GH, Ivanina AV, Matoo OB, Pörtner HO, Lannig G, et al (2012) Interactive effects of salinity and elevated CO_2_ levels on juvenile eastern oysters, *Crassostrea virginica* . J Exp Biol 215(1): 29–43. 22162851 10.1242/jeb.061481

[74] Hüning AK, Melzner F, Thomsen J, Gutowska MA, Krämer L, et al.. (2012) Impacts of seawater acidification on mantle gene expression patterns of the Baltic Sea blue mussel: implications for shell formation and energy metabolism. Mar Biol: 1–17.

[75] Liu W, Huang X, Lin J, He M (2012) Seawater acidification and elevated temperature affect gene expression patterns of the pearl oyster *Pinctada fucata* . PloS ONE 7(3): e33679. 22438983 10.1371/journal.pone.0033679 PMC3306283

[76] Melzner F, Stange P, Trübenbach K, Thomsen J, Casties I, et al (2011) Food supply and seawater pCO2 impact calcification and internal shell dissolution in the blue mussel *Mytilus edulis* . PloS ONE 6(9): e24223. 21949698 10.1371/journal.pone.0024223 PMC3174946

[77] Ries JB, Cohen AL, McCorkle DC (2009) Marine calcifiers exhibit mixed responses to CO_2_-induced ocean acidification. Geology 37(12): 1131–1134.

[78] McCulloch M, Falter J, Trotter J, Montagna P (2012) Coral resilience to ocean acidification and global warming through pH up-regulation. Nat Clim Chang advance online publication.

[79] McCulloch M, Trotter J, Montagna P, Falter J, Dunbar R, et al (2012) Resilience of cold-water scleractinian corals to ocean acidification: Boron isotopic systematics of pH and saturation state up-regulation. Geochim Cosmochim Acta 87: 21–34.

[80] Zippay MKL, Hofmann GE (2010) Effect of pH on gene expression and thermal tolerance of early life history stages of red abalone (*Haliotis rufescens*). J Shellfish Res 29(2): 429–439.

[81] Venn A, Tambutté E, Holcomb M, Laurent J, Allemand D, et al. (in press) Impact of seawater acidification on pH at the tissue-skeleton interface and calcification in reef corals. Proc Natl Acad Sci U S A. 10.1073/pnas.1216153110 PMC3562847 23277567

[82] Houlbrèque F, Ferrier-Pagès C (2009) Heterotrophy in tropical scleractinian corals. Biol Rev 84(1): 1–17. 19046402 10.1111/j.1469-185X.2008.00058.x

[83] Doney SC, Fabry VJ, Feely RA, Kleypas JA (2009) Ocean acidification: The other CO2 problem. Annu Rev Mar Sci 1(1): 169–192. 10.1146/annurev.marine.010908.163834 21141034

[84] Polovina JJ, Howell EA, Abecassis M (2008) Ocean's least productive waters are expanding. Geophys Res Lett 35(3): L03618.

[85] Stoecker DK, Johnson MD, de Vargas C, Not F (2009) Acquired phototrophy in aquatic protists. Aquat Microb Ecol 57: 279–310.

[86] Dupont S, Moya A, Bailly X (2012) Stable photosymbiotic relationship under CO_2_-induced acidification in the acoel worm *Symsagittifera roscoffensis* . PloS ONE 7(1): e29568. 22253736 10.1371/journal.pone.0029568 PMC3253794

[87] Towanda T, Thuesen EV (2012) Prolonged exposure to elevated CO_2_ promotes growth of the algal symbiont *Symbiodinium muscatinei* in the intertidal sea anemone *Anthopleura elegantissima* . Biology Open 1(7): 615–621. 23213455 10.1242/bio.2012521 PMC3507300

[88] Eisen MB, Spellman PT, Brown PO, Botstein D (1998) Cluster analysis and display of genome-wide expression patterns. Proc Natl Acad Sci U S A 95(25): 14863–14868. 9843981 10.1073/pnas.95.25.14863 PMC24541

[89] Gasch AP, Spellman PT, Kao CM, Carmel-Harel O, Eisen MB, et al (2000) Genomic expression programs in the response of yeast cells to environmental changes. Mol Biol Cell 11(12): 4241–4257. 11102521 10.1091/mbc.11.12.4241 PMC15070

[90] Warner JR (1999) The economics of ribosome biosynthesis in yeast. Trends Biochem Sci 24(11): 437–440. 10542411 10.1016/s0968-0004(99)01460-7

[91] Edge SE, Morgan MB, Gleason DF, Snell TW (2005) Development of a coral cDNA array to examine gene expression profiles in *Montastraea faveolata* exposed to environmental stress. Mar Pollut Bull 51(5–7): 507–523. 16115654 10.1016/j.marpolbul.2005.07.007

[92] Brulle F, Mitta G, Leroux R, Lemière S, Leprêtre A, et al (2007) The strong induction of metallothionein gene following cadmium exposure transiently affects the expression of many genes in *Eisenia fetida*: A trade-off mechanism? Comp Biochem Physiol C-Toxicol Pharmacol 144(4): 334–341. 17150412 10.1016/j.cbpc.2006.10.007

[93] Su J, Yang C, Xiong F, Wang Y, Zhu Z (2009) Toll-like receptor 4 signaling pathway can be triggered by grass carp reovirus and *Aeromonas hydrophila* infection in rare minnow *Gobiocypris rarus* . Fish Shellfish Immunol 27(1): 33–39. 19264133 10.1016/j.fsi.2009.02.016

[94] Wang B, Li F, Dong B, Zhang X, Zhang C, et al (2006) Discovery of the genes in response to White Spot Syndrome Virus (WSSV) infection in *Fenneropenaeus chinensis* through cDNA microarray. Mar Biotechnol 8(5): 491–500. 16794954 10.1007/s10126-005-6136-4

[95] Seo J, Lee KJ (2004) Post-translational modifications and their biological functions: proteomic analysis and systematic approaches. J Biochem Mol Biol 37(1): 35–44. 14761301 10.5483/bmbrep.2004.37.1.035

[96] Wong KKW, Lane AC, Leung PTY, Thiyagarajan V (2011) Response of larval barnacle proteome to CO_2_-driven seawater acidification. Comp Biochem Physiol D-Genomics Proteomics 6(3): 310–321. 10.1016/j.cbd.2011.07.001 21831737

[97] Venn A, Tambutté E, Holcomb M, Allemand D, Tambutté S (2011) Live tissue imaging shows reef corals elevate pH under their calcifying tissue relative to seawater. PloS ONE 6(5): e20013. 21637757 10.1371/journal.pone.0020013 PMC3103511

