# Supplementary material for: Genes Related to Ion-Transport and Energy Production Are Upregulated in Response to CO2-Driven pH Decrease in Corals: New Insights from Transcriptome Analysis
Source: PLoS One. 2013 Mar 27;8(3):e58652. doi: 10.1371/journal.pone.0058652 (PMC3609761; doi:10.1371/journal.pone.0058652)
Supplement: File S1 — Biological function, annotation, BlastX top hit species, BlastX evalue, contig number and primer sequence of the selected candidate genes. (DOCX) [file pone.0058652.s001.docx]

Supplementary File 1: Biological function, annotation, blast top hit species, contig number and primer sequence of the selected candidate genes

| Biological function | Annotation | BlastX top hit species | BlastX *e*value | Contig number | Primer |
| --- | --- | --- | --- | --- | --- |
| Calcification process | Voltage-gated calcium channel | *Brugia malayi* | 3.9E-13 | Locus_51519_Transcript_1 | F: attggtatgcacggacgaag  R: tccattccagttgtctccag |
|  | Sodium calcium exchanger | *Nematostella vectensis* | 5.7E-31 | transcripts_v2_44780 | F: agcaagtcagcccaaatgag  R: atccattccagcaccaagac |
|  | Plasma membrane calcium ATPase | *Stylophora pistillata* | 1.2E-41 | Locus_55224_Transcript_1 | F: gcaacagccatctgttctg  R: ctgtccttgttttggcacttc |
|  | HCO3- exchanger 3 | *Nematostella vectensis* | 1.8E-10 | Locus_72394_Transcript_1 | F: aaagtaccccggtcatttcg  R: tcaatcctggaggcatgaac |
|  | Sodium bicarbonate cotransporter 1 | *Oreochromis mossambicus* | 1.1E-10 | Locus_38592_Transcript_1 | F: accaccataccttgcacagtc  R: ttgcatgatagcccagtgac |
|  | Sodium bicarbonate cotransporter | *Strongylocentrotus purpuratus* | 1.8E-11 | Locus_62347_Transcript_1 | F: atcatggagatccggcaac  R: agccctgacgatgattttg |
|  | Na-dependent Cl/HCO3 exchanger | *Loligo pealei* | 2.7E-41 | Locus_57537_Transcript_1 | F: ggtttgatgggcacaaagac  R: aaacggaacggcatgtagtc |
|  | Sodium bicarbonate cotransporter | *Homo sapiens* | 2.6 E-103 | Locus_20518_Transcript_1 | F: cggcttactccaacgatttc  R: caccatcattgccaacagac |
|  | Sodium bicarbonate cotransporter | *Taenuipygia guttata* | 4.8E-60 | transcripts_v2_1213 | F: tggtgaagatgcaggaagtg  R: tctgcatcaccaggagtttg |
|  | Carbonic anhydrase I | *Stylophora pistillata* | 1.2 E-9 | Locus_42936_Transcript_1 | F: aactagccaaggaaaacagatcc  R: atcgcagctaaactggcttg |
|  | Carbonic anhydrase II | *Stylophora pistillata* | 9.3E-113 | transcripts_v2_567 | F: tccatagtcttggcacatgg  R: tccatagtcttggcacatgg |
|  | Galaxin-like1-like1 | *Acropora millepora* | 1.0 E-8 | Locus_74336_Transcript_1 | \| F: aagtgctgctacaacagagtgg \| \| --- \| \| R: acttgtgagtccacggattg \| |
|  | Galaxin-like1-like2 | *Acropora millepora* | 3.0E-16 | transcripts_v2_46613 | \| F: atatgcatcttgccctcgtc \| \| --- \| \| R: tacgcagtattccgtggatg \| |
|  | Galaxin-like | *Galaxea fascicularis* | 4.3 E-4 | Locus_74019_Transcript_1 | \| F: cgacgcaaagtttgatatgtg \| \| --- \| \| R: agacgatgttaccaccacagc \| |
|  | Bone morphogenetic protein 1 | *Branchostoma floridae* | 4.6 E-6 | Locus_69171_Transcript_1 | F: tcgttttcacctgctaccaa  R: cccaattttcccgacaacta |
|  | Bone morphogenetic protein 7 | *Nematostella vectensis* | 3.2E-10 | Locus_21318_Transcript_1 | F: tggaactaaacgcctcaagc  R: gtcagtttctgttcgccatc |
| Photosynthesis | Light-harvesting protein | *Symbiodinium sp* | 1.4 E-26 | Locus_72480_Transcript_1 | F: catggacgtgtctccatgtt  R: cacgtcggtgaacttgatct |
|  | Plasma membrane proton efflux p-type ATPase-like | *Aureococcus anophagefferens* | 3.6 E-14 | Locus_68104_Transcript_1 | F: cagccacttgatggtcgtaa  R: ctctcggcgtatcttcaagc |
|  | Photosystem II protein L | *Heterocapsa triquetra* | 2.7 E-17 | Locus_64216_Transcript_1 | F: gagaacagcaccgagaggac  R: actggcaacagcttggagat |
|  | Photosystem I subunit IV | *Heterocapsa triquetra* | 1.2 E-25 | Locus_62203_Transcript_1 | F: cttcgacccgagtcctactg  R: caaagccatttgtgttcacg |
|  | Photosystem I subunit XI | *Heterocapsa triquetra* | 1.2E-104 | transcripts_v2_34789 | F: gtggtgcaggagaaggagag  R: ccatacacctccgcaaaagt |
|  | Photosystem I subunit III | *Symbiodinium sp* | 4.7E-61 | Locus_56575_Transcript_1 | F: cacattccactcacctgtgg  R: aagaaggttgcccagatcaa |
|  | Photosystem II cp43 protein | *Symbiodinium sp* | 9.6E-44 | transcripts_v2_6035 | F: cttgagaggcttctgctgct  R: tcagccgtagctttatgtgc |
|  | Photosystem I P700 chlorophyll a apoprotein A2 | *Akashiwo sanquinea* | 3.6E-22 | transcripts_v2_26768 | F: aattcgcggttattctgcac  R: cctgccagtaacctctccaa |
| Glycolysis | Hexokinase | *Nematostella vectensis* | 3.1 E-10 | Locus_9609_Transcript_1 | F: aggttttgcagcgagtttgt  R: aggcttccatcaacagcaat |
|  | Aldolase | *Nematostella vectensis* | 4.6E-141 | Locus_6992_Transcript_1 | F: acgagtgactgagcgaggtt  R: atagtgcccgtgctttcatc |
|  | Glyceraldehyde-3-phosphate dehydrogenase | *Nematostella vectensis* | 8.6E-147 | transcripts_v2_13354 | F: cgcttgaagacaccaactga  R: ccagctttggcatcaaaaat |
|  | Enolase | *Nematostella vectensis* | 0.0E0 | transcripts_v2_65585 | F: atttgcaaagcaggagcagt  R: tttattcccagcatggcttc |
| Krebbs cycle | Citrate synthase | *Nematostella vectensis* | 6.7E-178 | transcripts_v2_34322 | F: acaaggtgggtggtatgagc  R: cagtggatccaggcaaagat |
|  | Aconitate hydratase | *Nematostella vectensis* | 2.4E-103 | Locus_3922_Transcript_1 | F: gtttggccagtcttcagctc  R: cagggtgttagagcggtgat |
|  | Isocitrate dehydrogenase | *Nematostella vectensis* | 1.7E-170 | transcripts_v2_70609 | F: gtcgtcaatggaccaggagt  R: gtgacacctccagtccctgt |
|  | Oxoglutarate dehydrogenase | *Anolis carolinensis* | 0.0E0 | Locus_409_Transcript_1 | F: ggactttcagccatcgtcat  R: catgctgaaaccaagctcaa |
|  | ATP-citrate synthase | *Nematostella vectensis* | 1.6E-25 | Locus_29110_Transcript_1 | F: ctcctccaacacccatgtct  R: caaaccctcctcattcatgtc |
|  | Succinate dehydrogenase | *Mus musculus* | 1.4E-18 | Locus_26352_Transcript_1 | F: aaattcccttggccactttt  R: tggactgggctctagcagtt |
|  | Fumarase | *Nematostella vectensis* | 1.9E-39 | Locus_6324_Transcript_1 | F: tcaagtcatgggcaatcaaa  R: tacacaggcatctcccatca |
|  | Malate dehydrogenase | *Nematostella vectensis* | 5.3E-131 | Locus_5898_Transcript_1 | F: gccatttggctttgtacgat  R: ggactcctgcagggataaca |
| Oxidative phosphorylation | NADH dehydrogenase | *Nematostella vectensis* | 2.3E-16 | Locus_46943_Transcript_1 | F: gacgaaggaaagcaagcaac  R: cggcaataaccaaattcctg |
|  | Succinate dehydrogenase | *Mus musculus* | 1.4E-18 | Locus_26352_Transcript_1 | F: aaattcccttggccactttt  R: tggactgggctctagcagtt |
|  | Ubiquinol-cytochrome C reductase complex | *Nematostella vectensis* | 1.8E-93 | Locus_8844_Transcript_2 | F: aatgaatggcgaaagtcagg  R: aacatcaccaatggctgaca |
|  | ATP synthase subunit beta | *Tribolium castaneum* | 0.0E0 | transcripts_v2_425 | F: ggtcgagggtagatcaccaa  R: agtgtttctggacccacagg |
| Lipolysis and beta oxidation | Triglyceride lipase | *Acropora millepora* | 5.4E-10 | Locus_27044_Transcript_1 | F: ttgaattctccctccatcca  R: ggttctggggaagttgatga |
|  | Acyl-CoA dehydrogenase | *Nematostella vectensis* | 7.8E-30 | Locus_67964_Transcript_1 | F: gatgtgggctgttttggtct  R: cagtgcatttggtgtgttga |
|  | Hydroxyacyl-CoA dehydrogenase/3-Ketoacyl-CoA thiolase/enoyl-CoA hydratase (trifunctional protein) | *Nematostella vectensis* | 0.0E0 | transcripts_v2_23540 | F: gtcaatatgggctggttgct  R: ttgcatgcctatcatgtgg |
|  | Hydroxyacyl-CoA dehydrogenase | *Nematostella vectensis* | 1.1E-107 | transcripts_v2_1920 | F: gcaacaagcttgggagaaag  R: atgggccaatcagatgtttc |
| Kinase activity | Tyrosine kinase | *Nasonia vitripensis* | 6.5E-8 | Locus_25628_Transcript_1 | F: ctcaaggctggtctattgc  R:ttcttcaacaagtccagctc |
|  | Serine/Threonine-protein kinase H1 | *Nematostella vectensis* | 2.4E-18 | Locus_62445_Transcript_1 | F: atgcgatccaagagttcacc  R: tgaagcacgattacattgttca |
|  | Tyrosine-protein kinase | *Trobolium castaneum* | 1.9E-7 | Locus_20203_Transcript_1 | F: gtcccttcgcgagtagactg  R: gggatttttccattcccttt |
|  | Serine/threonine-proteine kinase TBK1 | *Nematostella vectensis* | 5.0E-8 | transcripts_v2_25678 | F: atcgctcaggacaaccaact  R: acaccgatgaccagtccttc |
