# Supplementary material for: Genes Related to Ion-Transport and Energy Production Are Upregulated in Response to CO2-Driven pH Decrease in Corals: New Insights from Transcriptome Analysis
Source: PLoS One. 2013 Mar 27;8(3):e58652. doi: 10.1371/journal.pone.0058652 (PMC3609761; doi:10.1371/journal.pone.0058652)
Supplement: File S2 — Table results of the GO term enrichment analysis. (DOCX) [file pone.0058652.s002.docx]

Supplementary file 2: Table results of the GO term enrichment analysis

| Biological process upregulated | | | |
| --- | --- | --- | --- |
| Gene Ontolgy-ID | **Gene Ontology Term** | **Gene (n)** | ***p*-value** |
| GO:0006090 | pyruvate metabolic process | 4 | 1.9E-04 |
| GO:0006091 | generation of precursor metabolites and energy | 32 | 2.1E-04 |
| GO:0006094 | gluconeogenesis | 4 | 1.9E-04 |
| GO:0006412 | translation | 60 | 5.3E-09 |
| GO:0006810 | transport | 98 | 1.3E-04 |
| GO:0007160 | cell-matrix adhesion | 7 | 2.9E-04 |
| GO:0009058 | biosynthetic process | 96 | 3.0E-04 |
| GO:0009059 | macromolecule biosynthetic process | 68 | 1.7E-05 |
| GO:0009141 | nucleoside triphosphate metabolic process | 29 | 5.1E-05 |
| GO:0009144 | purine nucleoside triphosphate metabolic process | 28 | 1.1E-04 |
| GO:0009150 | purine ribonucleotide metabolic process | 29 | 1.5E-04 |
| GO:0009199 | ribonucleoside triphosphate metabolic process | 28 | 1.1E-04 |
| GO:0009205 | purine ribonucleoside triphosphate metabolic process | 28 | 1.1E-04 |
| GO:0009259 | ribonucleotide metabolic process | 29 | 2.4E-04 |
| GO:0010467 | gene expression | 66 | 3.1E-06 |
| GO:0015979 | photosynthesis | 23 | 7.7E-06 |
| GO:0022607 | cellular component assembly | 24 | 2.7E-06 |
| GO:0031589 | cell-substrate adhesion | 7 | 2.9E-04 |
| GO:0034621 | cellular macromolecular complex subunit organization | 25 | 4.1E-07 |
| GO:0034622 | cellular macromolecular complex assembly | 24 | 1.4E-07 |
| GO:0034645 | cellular macromolecule biosynthetic process | 68 | 1.4E-05 |
| GO:0043623 | cellular protein complex assembly | 16 | 5.4E-05 |
| GO:0043933 | macromolecular complex subunit organization | 25 | 3.0E-06 |
| GO:0044085 | cellular component biogenesis | 28 | 1.5E-06 |
| GO:0044249 | cellular biosynthetic process | 96 | 1.5E-05 |
| GO:0044281 | small molecule metabolic process | 81 | 2.1E-04 |
| GO:0046165 | alcohol biosynthetic process | 5 | 1.5E-04 |
| GO:0051179 | localization | 98 | 1.4E-04 |
| GO:0051234 | establishment of localization | 98 | 1.3E-04 |
| GO:0051258 | protein polymerization | 15 | 7.8E-05 |
| GO:0055114 | oxidation reduction | 75 | 1.3E-04 |
| GO:0065003 | macromolecular complex assembly | 24 | 1.5E-06 |
| Biological process downregulated | | | |
| GO:0044260 | cellular macromolecule metabolic process | 189 | 4.7E-06 |
