# Supplementary material for: Genes Related to Ion-Transport and Energy Production Are Upregulated in Response to CO2-Driven pH Decrease in Corals: New Insights from Transcriptome Analysis
Source: PLoS One. 2013 Mar 27;8(3):e58652. doi: 10.1371/journal.pone.0058652 (PMC3609761; doi:10.1371/journal.pone.0058652)
Supplement: File S3 — Numerical value corresponding to the qRT-PCR. Quantification was normalized by comparison with results for exposure to pH 8.1 (present seawater pH); the results are presented as a log2 fold change in expression. (DOCX) [file pone.0058652.s003.docx]

Supplementary file 3: Numerical value corresponding to the qRT-PCR. Quantification was normalized by comparison with results for exposure to pH 8.1 (present seawater pH); the results are presented as a log2 fold change in expression.

| **Gene** | **pH 8.1** | **pH7.8** | **pH7.4** | **pH7.2** |
| --- | --- | --- | --- | --- |
| Voltage-gated Ca channel-like | 0.00 | 1.26 | 2.11 | 1.02 |
| Na/Ca exchanger-like | 0.00 | -0.60 | 0.72 | -1.29 |
| Plamsa membrane Ca ATPase 1-like | 0.00 | 0.56 | 0.52 | -1.59 |
| HCO3-exchanger-like | 0.00 | 1.26 | 4.15 | -0.65 |
| Na/HCO3 cotransporter 1-like | 0.00 | 2.35 | 1.89 | -0.81 |
| Na/HCO3 cotransporter-like | 0.00 | 1.81 | 1.79 | -1.53 |
| Na dependent Cl/HCO3 exchanger-like | 0.00 | 1.76 | 1.30 | -1.56 |
| Na/HCO3 cotransporter-like | 0.00 | 1.65 | 1.02 | -1.93 |
| Na/HCO3 cotransporter-like | 0.00 | 1.54 | 0.60 | 0.04 |
| Carbonic anhydrase-1-like | 0.00 | 2.72 | 2.50 | 1.74 |
| Carbonic anhydrase-2-like | 0.00 | 1.33 | 0.89 | 0.12 |
| Galaxin like1-like1 | 0.00 | 2.04 | 6.13 | 2.64 |
| Galaxin like1-like2 | 0.00 | 3.01 | 4.70 | 4.81 |
| Galaxin-like | 0.00 | 0.10 | 1.82 | -0.22 |
| Bone morphogenetic protein 1-like | 0.00 | 1.16 | 1.54 | -1.42 |
| Bone morphogenetic protein 7-like | 0.00 | 0.95 | 1.35 | -0.64 |
| Light harvesting protein-like | 0.00 | 3.06 | 2.47 | 3.43 |
| PS II protein L-like | 0.00 | 2.85 | 1.76 | 2.69 |
| PS I subunit IV-like | 0.00 | 2.51 | 1.42 | 2.44 |
| Plasma membrane proton-efflux p-Type ATPase-like | 0.00 | 3.81 | 1.27 | 3.58 |
| PS I subunit XI-like | 0.00 | 3.72 | 0.89 | 3.30 |
| PS I subunit III-like | 0.00 | 1.92 | 0.41 | 2.29 |
| PS II cp43-like | 0.00 | 2.44 | 0.20 | 2.57 |
| PS I p700 chlorophyll a apoprotein a2-like | 0.00 | 3.49 | -0.54 | 3.70 |
| Hexokinase-like | 0.00 | 1.66 | -0.96 | 0.79 |
| Aldolase-like | 0.00 | 0.50 | 0.99 | -0.29 |
| Glyceraldehyde-3-phosphate dehydrogenase-like | 0.00 | 0.47 | 0.75 | 0.13 |
| Enolase-like | 0.00 | 2.74 | 0.29 | -1.08 |
| Citrate synthase-like | 0.00 | -0.63 | 0.44 | -0.87 |
| Aconitate hydratase-like | 0.00 | 0.65 | 0.95 | 0.77 |
| Isocitrate dehydrogenase-like | 0.00 | -0.02 | 0.41 | -1.75 |
| Oxoglutarate dehydrogenase-like | 0.00 | 0.75 | -1.48 | -0.48 |
| ATP-citrate synthase-like | 0.00 | 1.49 | 1.75 | 0.08 |
| Succinate dehydrogenase-like | 0.00 | 0.40 | 0.99 | -0.13 |
| Fumarase-like | 0.00 | 1.16 | -2.00 | 0.93 |
| Malate dehydrogenase-like | 0.00 | 0.29 | 0.80 | -1.24 |
| NADH dehydrogénase-like | 0.00 | 0.63 | 1.78 | 0.28 |
| Succinate dehydrogenase-like | 0.00 | 3.44 | 0.99 | -0.09 |
| Ubiquinol-cytochrome C reductase complex-like | 0.00 | 0.40 | 1.51 | 0.30 |
| ATP synthase beta subunit-like | 0.00 | 0.36 | 0.94 | -0.92 |
| Triglyceride lipase-like | 0.00 | 0.20 | 0.96 | -1.00 |
| Acyl-CoA dehydrogenase-like | 0.00 | 0.76 | 1.43 | -1.12 |
| 3-Ketoacyl-CoA thiolase-like | 0.00 | 2.58 | 0.63 | 1.27 |
| Hydroxyacyl-CoA dehydrogenase-like | 0.00 | 0.45 | 0.89 | 1.06 |
| Tyrosien kinase-like | 0.00 | 1.77 | -4.46 | -1.03 |
| Serine/Threonine kinase h1-like | 0.00 | 0.10 | -4.35 | -0.89 |
| Tyrosine kinase-like | 0.00 | 0.42 | -2.73 | -1.33 |
| Serine/threonine kinase-like | 0.00 | 2.77 | -0.68 | -0.91 |
